# A single-cycle, recombinant VSV platform Nipah vaccine cross-protects against Hendra virus in nonhuman primates

**DOI:** 10.1371/journal.ppat.1014646

**Published:** 2026-09-22

**Authors:** Declan D. Pigeaud, Viktoriya Borisevich, Jacquelyn Turcinovic, Krystle N. Agans, Daniel J. Deer, Mack B. Harrison, Rachel O’Toole, Natalie S. Dobias, Abhishek N. Prasad, Karla A. Fenton, Robert W. Cross, Thomas W. Geisbert

**Affiliations:** 1 Galveston National Laboratory, University of Texas Medical Branch, Galveston, Texas, United States of America; 2 Department of Microbiology and Immunology, University of Texas Medical Branch, Galveston, Texas, United States of America; University of Zurich: Universitat Zurich, SWITZERLAND

## Abstract

Nipah virus (NiV) and Hendra virus (HeV) are highly pathogenic paramyxoviruses that produce severe, often fatal disease in humans and animals. Zoonotic spillover of these henipaviruses from the Pteropid bat natural reservoir occurs near-annually in Southeast Asia and Oceania. Outbreaks of NiV disease frequently exceed case fatality rates of 75%, and person-to-person transmission makes controlling outbreaks in low-resource environments challenging. HeV is less transmissible between humans; however, the overall mortality rate is 57%. Approaches to human vaccine development have largely focused on NiV given the larger case burden, and immunogen selection has centered on display of the NiV attachment (G) or fusion (F) surface glycoproteins. However, experimental vaccines displaying these NiV antigens have failed to uniformly cross-protect against HeV disease in preclinical models. The HeV (G) antigen was shown to cross-protect against both HeV and NiV when delivered in a protein subunit form; however, attempts to utilize mRNA or canarypox vectors failed to achieve equivalent protection. We previously developed and evaluated a single-cycle recombinant vesicular stomatitis virus-vectored vaccine displaying the (G) glycoprotein of Nipah virus strain Bangladesh (NiV-B). This experimental vaccine (G*rVSV∆G-NiV-G) demonstrated ideal characteristics of rapid and durable protection against NiV-B challenge in nonhuman primates. In the present work, we show that the G*rVSV∆G-NiV-G vaccine cross-protects against lethal HeV challenge, with the protective response driven by a balance of both cell-mediated and humoral compartments.

## Author summary

Emerging viruses Nipah and Hendra were first identified as human pathogens in the 1990s and can cause severe, often fatal disease for which there are no approved medical countermeasures. Both viruses are found to naturally circulate in fruit bats, which can transmit the virus to amplifying hosts such as horses and pigs, presenting a risk to both human and veterinary health. Vaccine countermeasures to protect humans against either virus are not available. We previously showed that a single dose of a recombinant vesicular stomatitis virus (rVSV) vectored vaccine incorporating a Nipah glycoprotein antigen could rapidly protect animals from lethal Nipah disease. Hendra virus is closely related to Nipah, and an ideal vaccine countermeasure should offer protection against both pathogens. In the present work, we performed experiments to investigate whether a single dose of the rVSV Nipah vaccine could also protect against Hendra virus. We found that the rVSV Nipah vaccine protected nonhuman primates from lethal Hendra virus disease and that protection was likely due to a combined immune response involving both humoral and cellular components. Our study suggests that cross-protection against Hendra virus by vaccination with a Nipah antigen vaccine likely requires a T-cell response, which has previously not been described.

## Introduction

Emerging viruses Nipah (NiV) and Hendra (HeV) present a significant risk to human and animal health on a global scale. HeV emerged in Queensland, Australia in 1994 following a zoonotic spillover from the *Pteropus alecto* natural reservoir to an equine intermediate host, which amplified and transmitted the virus to humans, resulting in fatal disease [1]. NiV emerged in 1998 and 1999 in Malaysia and Singapore following spillover from Pteropid bats (likely *Pteropus medius*) to swine, which subsequently produced a large outbreak in humans with high mortality [2,3]. The economic implications of NiV emergence were drastic and produced economic losses exceeding US $500 million due to culling 45% of Malaysia’s swine population, which was a necessary response to end the outbreak [4]. Veterinary implications for HeV emergence were similar though not as drastic, as the initial outbreak of HeV occurred at stable facilities housing thoroughbred racehorses, leading to the massive disruption of the horse racing industry. Recent equine outbreaks of HeV have occurred in regions home to large populations of horses and veterinary facilities [5]. The large risk to humans and animals posed by infection of amplifying hosts resulted in efforts to develop veterinary vaccines. A highly effective, soluble HeV attachment glycoprotein (G) vaccine was approved for equine use in Australia in 2012 [6]. Various candidates are under development for protecting swine from NiV, but none have yet been approved and deployed by regulatory authorities in NiV-endemic countries [4].

HeV infection of humans occurs following contact with fluids of HeV-infected horses, and those in the veterinary profession or equine industry are at the highest risk. Human to human transmission of HeV has not been documented; however, transmission between laboratory animal species through direct contact or inoculation with infected fluids is possible. Following an incubation period ranging 5–21 days, HeV-infected patients exhibit nonspecific, febrile symptoms such as fever, myalgia, headache, cough, and exhaustion which can progress to fatal neurological (meningitis, encephalitis, coma) and respiratory (interstitial pneumonia, pulmonary edema) disease. HeV infection can also produce a recrudescent, fatal encephalitis following resolution of symptomatic disease and detectable viral RNA in the blood [7]. The case fatality rate of HeV infection in humans is 57% (4/7). Although no specific antiviral therapies are available for HeV, ribavirin was administered in early human cases but was later shown to have limited efficacy in experimental animal studies [8–10]. A monoclonal antibody therapeutic, m102.4, was developed, validated in multiple animal models, and has been deployed under compassionate use guidelines for individuals exposed to HeV in Australia and the United States [7,11]. The m102.4 therapeutic recently underwent phase I clinical trials and was found to be safe and well tolerated [12].

Transmission of NiV to humans is possible through several routes including contact with the contaminated fluids of infected animals such as pigs or bats, consumption of contaminated date palm sap, or through close contact with a symptomatic, NiV-infected patient. Person-to-person transmission of NiV has been documented in a number of outbreaks in hospital settings, underscoring the need for effective infection control protocols [13–17]. After exposure, NiV-infected patients develop a nonspecific febrile illness following an incubation period ranging from 5 to 14 days. In severe and fatal disease, individuals exhibit clinical signs including fever, cough, dyspnea, and vomiting, which in fatal cases, rapidly progressed to severe respiratory distress, seizures, hemiparesis, encephalitis, and coma [16,18]. As of January 2026, the overall case fatality rate for human NiV disease is approximately 60%, across all outbreaks [19]. As with HeV, there are no specific antiviral drugs available to treat NiV infection; however, nucleoside analogs ribavirin and remdesivir have been deployed in several outbreaks with marginal improvements in survival outcomes [20]. Additionally, several other mAbs have been evaluated against NiV and demonstrated efficacy in preclinical animal models [21–24].

Experimental vaccines to NiV and HeV tested in preclinical models have spanned a wide array of platforms and antigen combinations. Shortly after the emergence of HeV and NiV, soluble forms of the attachment G glycoproteins from each virus were tested as potential vaccines in a feline model of disease [25,26]. The results from this study revealed that the HeV G attachment glycoprotein produces a more potent cross-neutralizing antibody response than the NiV G antigen. The soluble HeV G vaccine (HeV-sG) was later evaluated in additional henipavirus animal models including ferrets and nonhuman primates and was found to completely protect against both viruses [27–30]. However, equivalent efficacy of the HeV G antigen was not observed in viral vectored platforms for cross-protection or even homologous protection for an mRNA-LNP formulation [31–33]. We previously developed and evaluated a single-cycle, recombinant vesicular stomatitis virus (G*rVSV∆G) vaccine displaying the full-length G glycoprotein of the Bangladesh strain of NiV (NiV-B) [34]. The G*rVSV∆G-NiV-G vaccine provided uniform protection against a lethal challenge of NiV-B in multiple stringent animal models of NiV disease, and its utility as a countermeasure against NiV was demonstrated by studies investigating the onset and durability of protection. A single dose of the G*rVSV∆G-NiV-G vaccine completely protected against severe or lethal NiV disease in African green monkeys (AGM) vaccinated 7 days or over one year prior to challenge [35,36]. Protection against NiV-B disease was associated with high titers of neutralizing antibodies, which has been determined to correlate with the protection offered by the HeV-sG vaccine [37].

In the present study, we sought to determine whether the antigenic similarity of the G glycoproteins of NiV and HeV was sufficient for a single dose of the G*rVSV∆G-NiV-G to protect against heterologous HeV challenge. We performed two experiments, the first of which utilized the gold-standard AGM model of HeV disease to determine the level of protection afforded by a single dose of the G*rVSV∆G-NiV-G vaccine; and a second experiment, employing Syrian golden hamster (SGH) models of NiV and HeV to investigate potential mechanisms of cross-protection via passive immunization. Analysis of the humoral and inferred cell-mediated immune response through serology and targeted transcriptomics were conducted. We identified that a single, 1.0E7 PFU intramuscular dose of the G*rVSV∆G-NiV-G vaccine delivered 28 days prior to stringent HeV-g1 challenge conferred protection against lethal HeV disease, despite the absence of potent serum neutralizing antibody titers at the time of challenge. In passive transfer experiments we found that treatment with a single dose of serum from G*rVSV∆G-NiV-G vaccinated hamsters failed to protect against HeV-g1 challenge, but this treatment provided complete protection against homologous NiV-B challenge.

## Methods

### Ethics statement

All study protocols described in this manuscript were approved by the University of Texas Medical Branch (UTMB) Institutional Animal Care and Use Committee (IACUC), which were compliant with UTMB Institutional Biosafety Committee guidelines under biosafety level four containment. Animal facilities used in this work are accredited by the Association for the Assessment and Accreditation of Laboratory Animal Care International and adhere to principles specified in the eighth edition of the Guide for the Care and Use of Laboratory Animals, National Research Council.

### Cells and viruses

Baby hamster kidney clone WI-2 cells (BHK-WI2) used for vaccine preparation and titration were obtained from Kerafast (EH1011) and grow in DMEM (Gibco, cat# 11965092) supplemented with 5% heat-inactivated FBS (Gibco, A5669801), 1% glutaMAX (Gibco, 35050061) and 1% Pen/Strep (Gibco, 15070063). Vero76 cells (ATCC, CRL1587) used for titration of NiV-B and HeV-g1 were obtained from the American Type Culture Collection and maintained in EMEM (ATCC, 30–2003) containing 10% HI-FBS, Pen/Strep, and glutaMAX.

The HeV-g1 stock is derived from the HeV/Australia/Horse/2008/Redlands isolate (NCBI accession HM044317) and was obtained from the European Virus Archive (EVAg, 023V-02990). The challenge virus stock was passaged five times on Vero76 cells. The NiV strain Bangladesh stock used for challenge experiments and *in vitro* neutralization assays was derived from fatal human isolate 200401066. The challenge material was passaged twice on Vero E6 cells as previously described [35]. The G*rVSV∆G-NiV-G vaccine used for vaccination and challenge experiments was identical to the vaccine utilized in previous experiments [35,36]. No detectable mycoplasma or endotoxin contamination was present in viral challenge stocks or the G*rVSV∆G-NiV-G vaccine.

The HeV-g1 and NiV-B challenge viruses along with the G*rVSV∆G-NiV-G vaccine were deep sequenced via utilizing the Illumina platform to confirm molecular identity. Reads aligning to the AGM genome were discarded (genome assembly ChlSab1; Bowtie2 v2.3.4.3 [38]), and reads that did not align were metagenomically classified with Kraken2 v2.0.7-beta [39] to detect adventitious agents and other contaminants. All non-host reads were then aligned to the appropriate viral reference with Bowtie2 v2.3.4.3 [38] (NCBI AY988601.1). PCR duplicates were masked before calculating coverage, assembling a consensus, and calling single nucleotide variants. Alignment processing, duplicate masking, and consensus building were accomplished via Samtools v1.20 [40]. SNVs were called with LoFreq v2.1.3.1 [41] and annotated in R v4.4.0 using the Biostrings [42] library. No nonsynonymous mutations were detected at >5% relative abundance for either challenge stocks or the G*rVSV∆G-NiV-G vaccine.

### Nonhuman primate vaccination and challenge

A total of n = 8 healthy, adult African green monkeys (AGM, *Chlorocebus aethiops*, PreLabs, Lehigh Acres, FL) weighing ~ 2.9 to 7.3 kg) were randomized into two groups containing n = 5 (vaccinated) and n = 3 (naïve control). Animals were anesthetized on study day 0 (-28 DPI) with an intramuscular (i.m.) injection of ketamine and n = 5 were subsequently vaccinated i.m. with 1.0E7 PFU of G*rVSV∆G-NiV-G in the quadricep. The n = 3 positive controls received no vaccine. At study day 28 (0 DPI), all AGMs were anesthetized as described above and inoculated with a lethal dose of HeV-g1 or NiV-B via the combined it/in route depending on study arm. Vaccines and challenge inoculums were diluted to target doses in sterile HBSS containing 2% HI-FBS. The duration of the challenge study was 35 days. Following NiV-B challenge, all AGMs were monitored daily and scored for disease progression in accordance with henipavirus human endpoint scoring criteria approved by the UTMB Institutional Animal Care and Use Committee (IACUC). The UTMB facilities used for this work are accredited by the Association for Assessment and Accreditation of Laboratory Animal Care International (AAALAC) and adhere to the principles specified in the National Research Council Guide for the Care and Use of Laboratory Animals. The clinical scoring criteria assess numerous parameters which include posture, activity level, behavior, food intake, respiration, and henipavirus-disease manifestations such as central nervous system disorders. A score of ≥ 9 indicated that an animal met the criteria for euthanasia.

### Syrian golden hamster vaccination, passive transfer, and challenge

A total of n = 62 female, six to eight-week-old Syrian golden hamsters (SGH) were obtained from Envigo for immunization, passive transfer, and challenge experiments. All hamsters were randomized into experimental groups and housed in HEPA-filtered caging containing bedding material, destructive enrichment, and food/water available *ad libidum*. Animals were anesthetized via inhalational isoflurane for all procedures. All SGHs were observed for clinical signs of disease by experienced husbandry and veterinary staff and scored for clinical disease in accordance with parameters approved by the UTMB IACUC. Subjects that met the clinical scoring criteria or reached the scientific endpoint of the studies were euthanized following institutionally approved methods.

Antiserum for the passive transfer and challenge experiment was generated by vaccinating two groups of SGHs (n = 15 each) via the i.m. route with 1.0E7 PFU of G*rVSV∆G-NiV-G or G*rVSV∆G-GFP vaccine on study day 0. Animals were monitored for clinical signs following vaccine administration and subsequently humanely euthanized on study day 28 following blood collection into clot-activator serum-separator tubes. Serum was separated by centrifugation and 200 µL aliquots were prepared for downstream immunoassays. The remaining volume of sera for each subject within each vaccine group was pooled and stored at -80°C for subsequent use in the passive transfer experiment.

To evaluate the capacity for anti-G*rVSV∆G-NiV-G antibodies to protect against NiV or HeV challenge a total of n = 32 SGHs were randomized into four groups containing n = 8 subjects each. On study day -1, all subjects were anesthetized and treated intraperitoneally (i.p.) with 0.5 mL of anti-G*rVSV∆G-NiV-G (specifically treated) or anti-G*rVSV∆G-GFP (control) antiserum pools. After 24 hours, on study day 0, subjects were challenged i.p. with a 1.0E4 PFU target dose of HeV-g1 or NiV-B. Following challenge, subjects were monitored for clinical signs of henipavirus disease until the study endpoint (day 28) at which point any survivors were humanely euthanized. Oral swabs were collected at the time of challenge, at 3, 5, 7, 10, and 14 days post infection (DPI), and at terminal events. Tissues and serum were samples at terminal events and the study endpoint.

### Hematology and serum biochemistry

Total white blood cell counts, white blood cell differentials, red blood cell counts, platelet counts, hematocrit values, total hemoglobin concentrations, mean cell volumes, mean corpuscular volumes, and mean corpuscular hemoglobin concentrations were analyzed from blood collected in tubes containing EDTA using a Vetscan HM5 laser based hematologic analyzer (Zoetis). Serum samples were tested for concentrations of albumin, amylase, alanine aminotransferase (ALT), aspartate aminotransferase (AST), alkaline phosphatase (ALP), blood urea nitrogen (BUN), calcium, creatinine (CRE), C-reactive protein (CRP), gamma-glutamyl transferase (GGT), glucose, total protein, and uric acid by using a Piccolo point-of-care analyzer and Biochemistry Panel Plus analyzer discs (Abaxis).

### Histopathology and immunohistochemistry

Necropsy was performed on all subjects. Tissue samples of all major organs were collected for histopathologic and immunohistochemistry (IHC) examination, immersion-fixed in 10% neutral buffered formalin, and processed for histopathology. Relative severity scores for histological lesions and immunoreactivity were assigned by an American College of Veterinary Pathologists board-certified veterinary pathologist (Table 1 in S1 Appendix). Representative photomicrographs were qualitatively considered to display lesions that were nominally or ordinally measured by masking of the veterinary pathologist after examination and ranking lesions to satiate the study objectives.

Tissue sections were deparaffinized and rehydrated through xylene and graded ethanols. Slides went through heat antigen retrieval in a steamer at 95°C for 20 minutes in Sigma Citrate Buffer, pH 6.0, 10x (Sigma Aldrich, St. Louis, MO). The tissue sections were processed for IHC using the Thermo Autostainer 360 (ThermoFisher, Kalamazoo, MI). Specific anti-henipavirus nucleoprotein (N) immunoreactivity was detected using an anti-Henipavirus N primary antibody at a 1:4000 dilution for 60 minutes. Secondary antibody used was biotinylated goat anti-rabbit IgG (Vector Laboratories, Burlingame, CA #BA-1000) at 1:200 for 30 minutes followed by Vector Streptavidin Alkaline Phosphatase at a dilution of 1:200 for 15 minutes (Vector Laboratories #SA-5100). Slides were developed with ImmPact Red Substrate Kit (Vector Laboratories #SK-5105) for 20 minutes and counterstained with hematoxylin for 30 seconds.

### Plaque assay

Replicating HeV-g1 or NiV-B was detected from plasma, tissue, and swab specimens by standardized plaque assay on Vero76 cells. Briefly, samples were diluted in EMEM containing 2% heat-inactivated FBS (HI-FBS) and adsorbed in duplicate on confluent cell monolayers in 6-well plates for 1 hour at 37°C. Sample wells were overlayed with 0.8% agarose in 2X MEM salts and allowed to incubate at 37°C, 5% CO_2_ for 48 hours (HeV-g1) or 72 hours (NiV-B). Plates were stained with 5% neutral red solution in PBS supplemented with 5% HI-FBS for 24 hours at 37°C, 5% CO_2_, after which stain was decanted and plaques were quantified.

### Quantification of viral genomes in blood and tissue samples

Total RNA was extracted from blood, tissue, and swab specimens using Qiagen QIAmp Viral RNA Mini (Qiagen #52904) and Qiagen RNeasy (Qiagen #74104) kits following manufacturer’s instructions. Quantitative reverse transcriptase-polymerase chain reaction (qRT-PCR) using primers/probe targeting the (F) gene of HeV-g1 or the (N) gene of NiV-B was performed to determine the quantity of vRNA present in the samples. The probe sequences utilized for detection of HeV-g1 and NiV-B are 6FAM-5’CTCGGCTGTATCTGGAGCAGTCAC-3’-TAMRA (HeV-g1) and 6FAM-5’-CTGCAGGAGGTGTGCTCATCGGAGG-3’-TAMRA (NiV-B) and were obtained from Invitrogen. Primer sequences are available upon request. Cycle threshold (Ct) values representing copies of viral genomes were used to calculate genome equivalents (GEq) of HeV-g1 or NiV-B based on a six-point standard curve consisting of serial dilutions of purified vRNA extracted from challenge virus stocks. Viral genome equivalents were calculated using Avogadro’s number and the molecular weight of the respective viral genomes. The limit of detection (LOD) for each qRT-PCR assay for detection of HeV-g1 or NiV-B is 1000 GEq/mL or g of tissue.

### Virus neutralization assays

The neutralizing titer of EDTA plasma specimens from AGMs or serum from SGHs at selected timepoints was determined by standard plaque reduction neutralization test (PRNT). Plasma samples were serially diluted and incubated with ~100 PFU of HeV-g1 or NiV-B for 1 hour at 37°C. Sample dilution media (EMEM supplemented with 2% HI-FBS) was included alongside the vaccinated or treated samples as an assay control. Individual samples were plated in duplicate, and titers were determined as previously described [43]. Percent neutralization was calculated by comparing the plaque counts of samples at each dilution to assay controls.

### ELISA

IgG titers to HeV or NiV G antigen in plasma specimens collected from G*rVSV∆G-NiV-G vaccinated AGMs were assayed using a nonhuman primate species ELISA kit from Alpha Diagnostic International (RV-501120–1, NIV-015) according to manufacturer’s instructions. Anti-HeV G or NiV G IgG antibody titers from serum samples collected from SGHs were assayed utilizing the HeV or NiV G antigen coated plates from the Alpha Diagnostic International kits. Briefly, serum samples were serially diluted in 1X PBS supplemented with 0.05% Tween20 and 2% BSA and plated and subsequently incubated for 1 hour at room temperature. Plates were then decanted and washed four times with 1X PBS supplemented with 0.05% Tween20. The HRP-conjugated goat-anti Syrian Hamster IgG secondary antibody (Invitrogen #PA1–28823) was diluted 1:10,000 in sample diluent buffer and incubated at room temperature for 1 hour. Plates were then decanted and washed as described above. SIGMAFAST OPD substrate (Millipore-Sigma, #P9187) was prepared according to manufacturer instructions and added to wells. Plates were allowed to develop for 10 minutes at room temperature in the dark before being stopped with 3M sulfuric acid. For both NHP and SGH assays, plates were imaged on a BioTek Cytation 5 multimode plate reader and all samples were assayed in duplicate. Endpoint titers were defined as the last reciprocal dilution of serum samples with an absorbance value of ≥0.150 after subtracting blank well values.

### Targeted transcriptomics

Whole blood samples collected from AGMs containing HeV-g1 were inactivated in AVL buffer (Qiagen) prior to removal from BSL-4 containment. RNA was extracted in accordance with manufacturer’s protocols utilizing the QIAcube automated molecular extraction system (Qiagen). Purified RNA was hybridized with the NHP_Immunology_V2 reporter and capture codesets containing ~779 targets (Bruker Spatial Biology) for ~24h at 65˚C prior to being loaded into nCounter microfluidic cartridges. Cartridges were immediately run on a Nanostring nCounter SPRINT Profiler (Bruker Spatial Biology) to measure fluorescent counts for each gene target. Output RCC files were loaded into nSolver v4.0 for QC and thresholded count matrices were exported for samples that passed assay quality controls. Thresholded count data matrices were analyzed in R v4.5.0 using limma v3.65.3 [44].

Three differential expression (DE) analyses were performed. First, the host response in unvaccinated controls was evaluated by comparing 4 DPI and terminal (6–7 DPI) to baseline (0 DPI), binning by days postinfection. Second, the host response in vaccinated animals was evaluated by comparing 4, 7, and 10 DPI samples to baseline. Finally, vaccinated animals were compared to controls at baseline, 4 DPI, and 7 DPI/terminal to quantify diverging host response patterns. For each comparison, significantly DE genes were determined by an FDR-adjusted p-value <0.05 and a log_2_ fold change >1 or <-1.

Digital cell type deconvolution was performed on all Nanostring-profiled samples via CIBERSORTx [45] run in absolute mode using the LM22 signature matrix, B-mode batch correction, 1000 permutations, and the CPM matrix as input. Significance comparisons in control and vaccinated time-series analyses were performed for each cell type via Friedman rank-sum test with post-hoc Dunn’s test. FDR-adjusted p-values <0.05 were considered significant. Significance comparisons between treatment groups (e.g., controls at 0 DPI vs. vaccinated at 0 DPI) were performed via Mann-Whitney U-test for each cell type, with p-values <0.05 considered significant. Statistical comparisons were performed via rstatix v0.7.2 in R v4.5.0.

## Results

### Survival, clinical disease, and viremia of G*rVSV∆G-NiV-G vaccinated AGMs challenged with HeV-g1

Five healthy, adult AGMs were vaccinated with a single 1.0E7 PFU dose of the G*rVSV∆G-NiV-G vaccine via the i.m. route with three subjects serving as unvaccinated (naïve) positive controls. At 28 days post- vaccination, (0 DPI) all n = 8 AGMs were challenged with a lethal dose (5.0E5 PFU) of HeV-g1 isolate HeV/Australia/Horse/2008/Redlands via the combined intratracheal/intranasal (i.t./i.n.) route and subsequently monitored for clinical signs of disease. All G*rVSV∆G-NiV-G vaccinated AGMs (NiVG-1 to NiVG-5) survived until the predetermined scientific endpoint of the study at 35 DPI (Fig 1A), while the n = 3 positive control subjects (HeV-C1 to HeV-C3) developed clinical signs of severe HeV disease and met clinical endpoints requiring humane euthanasia at 6 and 7 DPI, respectively (Fig 1B). A single G*rVSV∆G-NiV-G vaccinated subject (NiVG-3) exhibited mild clinical signs at 6, 7, and 9 DPI but subsequently recovered and remained clinical well until the scientific endpoint of the study (Fig 1B). A transient increase in respiration rates was observed for both NiVG-3 and NiVG-4 at 7 DPI but began to decrease by 8 DPI and returned to baseline levels by 10 DPI (Fig 1C). In contrast, all three unvaccinated positive control subjects developed severe respiratory clinical signs accompanied with substantial increases in respiration rates which continued to increase during acute and terminal stages of disease (Fig 1C). Additional clinical signs in the positive control subjects included unresponsiveness, weakness, hypersalivation, and recumbency (Table 1).

**Table 1:** Clinical description and outcomes of VSV-NiV-vaccinated AGMs following challenge with HeV-g1.

Subject	Sex	Clinical illness	Clinical pathology
NiVG-1	M	None. Subject survived to study endpoint (d35).	Lymphocytopenia (d7); monocytopenia (d21, 28, 35); eosinopenia (d4); basopenia (d4); neutrophilia (d7); eosinophilia (d7); basophilia (d7, 14); CRP ↑ (d4).
NiVG-2	F	Decreased appetite (d1, 5–8, 11, 15, 16, 21–23, 29–31, 34, 35); hypothermia (d10). Subject survived to study endpoint (d35).	Lymphocytopenia (d4); thrombocytopenia (d7, 35); neutropenia (d4, 10, 21, 28, 35); eosinopenia (d4, 7, 10, 28, 35); basopenia (d4, 7, 10, 28, 35); monocytosis (d35); CRE ↑ (d7).
NiVG-3	M	Tachypnea (d6, 9). Subject survived to study endpoint (d35).	Lymphocytosis (d14); monocytosis (d7, 10, 14, 21); neutrophilia (d10, 14); eosinophilia (d14); basophilia (d10, 14); hypoamylasemia (d7); CRP ↑ (d7).
NiVG-4	F	Tachypnea (d7, 9); fever (d7). Subject survived to study endpoint (d35).	Thrombocytopenia (d7); neutropenia (d4, 7, 10, 14, 21, 35); eosinopenia (d35); basopenia (d4, 35); lymphocytosis (d10, 14); monocytosis (d10, 14, 35); hypoamylasemia (d4, 21, 28).
NiVG-5	F	Decreased appetite (d8, 14, 15, 26, 28, 29, 34). Subject survived to study endpoint (d35).	Monocytopenia (d4, 7, 28, 35); neutropenia (d4, 7, 10, 14, 21, 28, 35); eosinopenia (d4, 7, 10, 14, 21, 28, 35); basopenia (d4, 7, 21, 28, 35); lymphocytosis (d4, 10, 14, 21, 28, 35); monocytosis (d14).
HeV-C1	M	Decreased appetite (d4); anorexia (d6); depression (d6); hunched posture (d6); tachypnea (d6); hypersalivation w/ blood (d6); recumbency (d6). Subject euthanized (d6).	Lymphocytopenia (d4); thrombocytopenia (d6); eosinopenia (d4); basopenia (d4, 6); monocytosis (d6); neutrophilia (d6); hyperglycemia (d6); hypoalbuminemia (d6); hypoproteinemia (d6); CRE ↑ (d6); AST ↑ (d6); CRP ↑ (d4, 6).
HeV-C2	F	Decreased appetite (d1); anorexia (d5-7); tachypnea (d6, 7); hunched posture (d7). Subject euthanized (d7).	Lymphocytopenia (d4, 7); thrombocytopenia (d7); monocytopenia (d4, 7); eosinopenia (d4, 7); basopenia (d4, 7); CRP ↑ (d7).
HeV-C3	F	Decreased appetite (d1, 5, 7); tachypnea (d7); weakness (d7); recumbency (d7); unresponsiveness (d7). Subject euthanized (d7).	Lymphocytopenia (d4, 7); thrombocytopenia (d7); eosinopenia (d4); basopenia (d4, 7); AST ↑ (d7); CRP ↑ (d7).

Days after HeV-g1 challenge are in parentheses. All reported findings are in comparison to baseline (day of challenge [d0]/d28 post-vaccination) values. Decreased appetite is defined as ≤ 65% of food consumed from the previous day. Anorexia is defined as no food consumed from the previous day. Fever is defined as a temperature more than 2.5 °F over baseline, or at least 1.5 °F over baseline and ≥ 103.5 °F. Hypothermia is defined as a temperature ≤3.5°F below baseline. Lymphocytopenia, monocytopenia, erythrocytopenia, thrombocytopenia, neutropenia, eosinopenia, and basopenia are defined by a ≥ 35% drop in numbers of lymphocytes, monocytes, erythrocytes, platelets, neutrophils, eosinophils, or basophils, respectively. Lymphocytosis, monocytosis, neutrophilia, eosinophila, and basophilia are defined by a 100% or greater increase in numbers of lymphocytes, monocytes, neutrophils, eosinophils, and basophils, respectively. Hyperglycemia is defined as a 100% or greater increase in levels of glucose. Hypoglycemia is defined by a ≥ 25% decrease in levels of glucose. Anemia is defined as a concurrent ≥25% decrease in erythrocyte count, Hct, and Hgb. Hypoalbuminemia is defined by a ≥ 25% decrease in levels of albumin. Hypoproteinemia is defined by a ≥ 25% decrease in levels of total protein. Hypoamylasemia is defined by a ≥ 25% decrease in levels of serum amylase. Hypocalcemia is defined by a ≥ 25% decrease in levels of serum calcium. Increases in ALT, AST, ALP, CRE, CRP, Hct, and Hgb were graded on the following scale:  ↑ = 1–5 fold, ↑↑ = > 5–10 fold, ↑↑↑ = > 10–20 fold, ↑↑↑↑ = > 20-fold, ↓ = ≥ 50% decrease. (BUN) blood urea nitrogen, (ALT) alanine aminotransferase, (AST) aspartate aminotransferase, (ALP) alkaline phosphatase, (CRE) Creatinine, (CRP) C-reactive protein, (Hct) hematocrit, (Hgb) hemoglobin.

**Fig 1:**
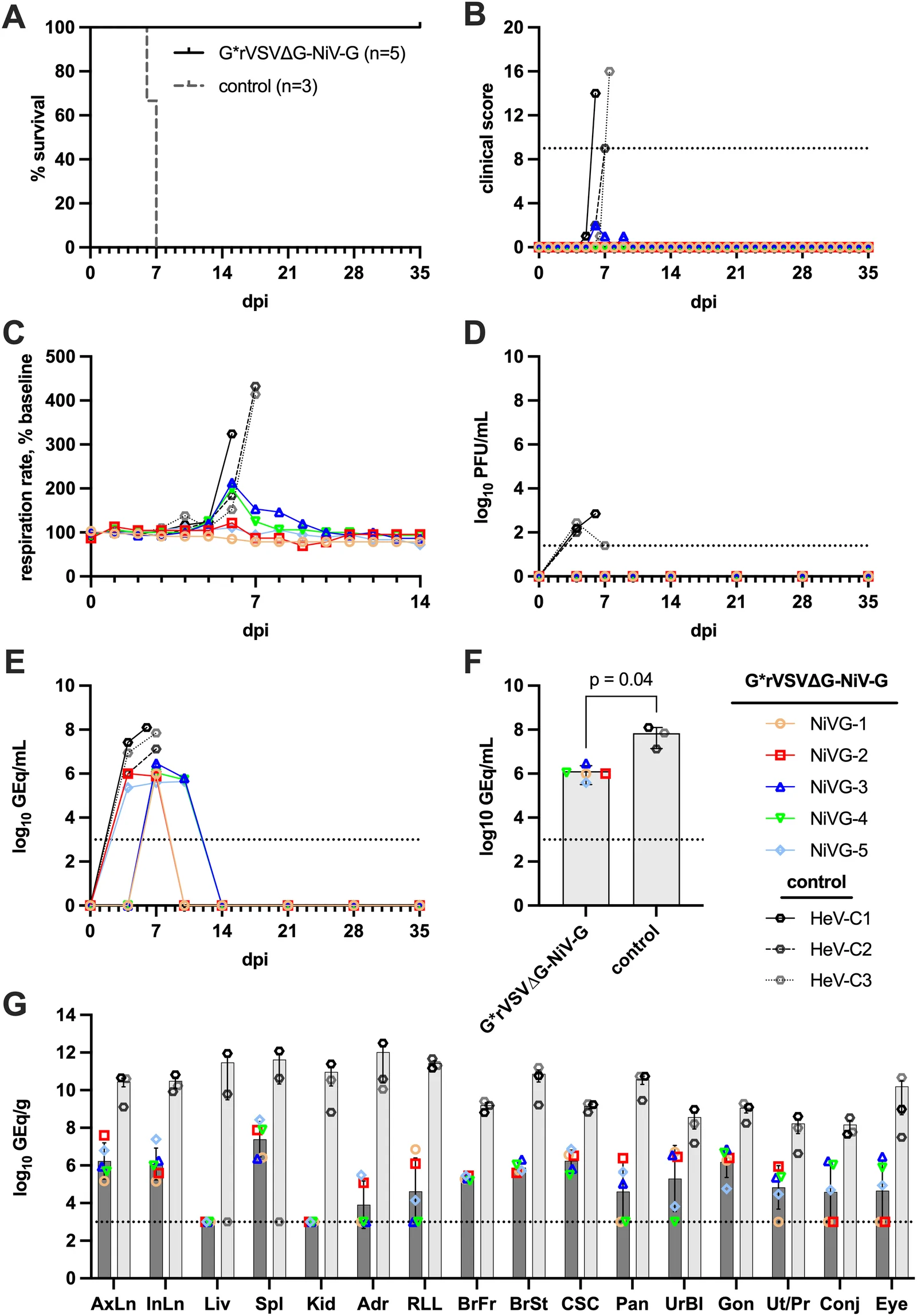
Survival, clinical disease, and viremia observed in G*rVSV∆G-NiV-G vaccinated or unvaccinated positive control AGMs following challenge with HeV-g1. A) Percent survival of vaccinated (solid black line) or unvaccinated control (dashed grey line) AGMs following HeV-g1 challenge until the study endpoint at 35 DPI, significant difference (p = 0.0067) between survival curves was identified by Logrank (Mantel-Cox) test for survival proportions. B) Quantitative clinical score of AGMs following HeV-g1 challenge, dotted line represents clinical endpoint requiring euthanasia. C) Respiration rates represented as percent of baseline for vaccinated and control AGMs from 0 to 14 DPI. The mean of respiration rates observed at 0 and 1 DPI were used as the baseline values. D) Detection of replicating HeV-g1 in plasma of infected AGMs by plaque assay. E) Detection of HeV-g1 viral genomes by qRT-PCR in whole blood specimens of HeV-g1 infected subjects. F) Comparison of peak HeV-g1 vRNA detected by qRT-PCR in vaccinated and control subjects. A 2-tailed Kolmogorov-Smirnov test was performed to determine significant differences between the vRNA load distribution between G*rVSV∆G-NiV-G and naïve control groups. G) HeV-g1 viral genomes detected in tissue specimens collected at euthanasia (35 DPI for vaccinated subjects, terminal timepoints for controls). For all plots, individual points represent the mean of two technical replicates. Horizontal lines indicate the mean, error bars indicate ±SD. Dotted horizontal line indicates the LOD for the specified assay, 999 GEq/mL for qRT-PCR and 25 PFU/mL for plaque assay. Samples with no detectable vRNA or titer were plotted below the LOD (1 GEq/mL and 999 GEq/g, or 1 PFU/mL for qRT-PCR and plaque assays, respectively). AxLn, axillary lymph node; IngLn, inguinal lymph node; Liv, liver; Spl, spleen; Kid, kidney; Adr, adrenal gland; RLL, right lower lung lobe; BrFr, brain frontal; BrSt, brain stem; CSC, cervical spinal cord; Pan, pancreas; UrBl, urinary bladder; Gon, gonad; Ut/Pr, uterus or prostrate; Conj, conjunctiva.

Clinical pathology assays were performed on whole blood and serum specimens collected at predetermined sampling timepoints and terminal endpoints to identify hematological and biochemical abnormalities (Table 1). All vaccinated subjects exhibited some decreases in white blood cell counts following challenge, and (2/5) subjects developed thrombocytopenia at 7 DPI. All positive control subjects developed hematological abnormalities characteristic of severe HeV disease including lymphocytopenia and thrombocytopenia. Increases in serum levels of c-reactive protein, an acute phase marker associated with systemic inflammation, was elevated in 2/5 vaccinated subjects at 4 or 7 DPI but subsequently returned to baseline levels (Table 1).

Replicating HeV-g1 was assayed in plasma samples by plaque assay following challenge, and titers were not detected at any point in the G*rVSV∆G-NiV-G vaccinated group (Fig 1D). HeV-g1 was detected in the plasma of all positive controls (n = 3) starting at 4 DPI, and titers ranged from 2.0 – 2.4 log10 PFU/mL. The peak viral load of any control subject was detected in HeV-C1 at 6 DPI, at 2.8 log10 PFU/mL. HeV-g1 viral genomes (vRNA) were detected and quantified by qRT-PCR performed on RNA extracted from whole blood samples post-challenge (Fig 1E). All vaccinated subjects had detectable vRNA for at least one sampling timepoint, and the vRNA load for vaccinated subjects ranged from 5.4 – 6.5 log10 GEq/mL. All positive control subjects had detectable HeV-g1 vRNA by 4 DPI, ranging from 6.0 – 7.4 log10 GEq/mL, which peaked at terminal timepoints. Peak vRNA levels were compared between the specifically vaccinated and positive control groups, with control animals exhibiting higher peak circulating viral RNA (Fig 1F). The mean of peak vRNA for the G*rVSV∆G-NiV-G cohort was 6.1 log10 GEq/mL compared to 7.8 log10 GEq/mL for unvaccinated positive controls.

HeV-g1 viral genome equivalents were detected by qRT-PCR in tissues sampled from G*rVSV∆G-NiV-G-vaccinated and positive control AGMs (Fig 1G). HeV-g1 vRNA was detected in the vaccinated subjects across all lymphoid tissue samples assayed (mean 7.5 log10 GEq/g, range 6.6 – 8.4 log10 GEq/g). The highest vRNA burden of select lymphoid tissues for vaccinated animals was found in the spleen, averaging 7.4 log10 GEq/g. High levels of vRNA were observed in all tissues sampled from positive control subjects, except for splenic tissue of HeV-C3, which did not have detectable vRNA. The mean level of vRNA in the spleen tissue for the remaining n = 2 controls was 11.4 log10 GEq/g. In respiratory tissues sampled, 4/5 vaccinated AGMs had detectable viral genomes in at least 1 lung lobe (Fig 1 in S1 Appendix), with the mean vRNA load being 4.6 log10 GEq/g and ranging from 3.9 – 7.7 log10 GEq/g (Fig 1G). In the positive controls, HeV-g1 vRNA was detected in all respiratory tissues sampled, averaging 11.7 GEq/g (range 10.7 – 12.7 log10 GEq/g). In the central nervous system (CNS) tissues, all vaccinated and positive control subjects had detectable HeV-g1 vRNA; however, quantities were much lower in G*rVSV∆G-NiV-G vaccinated AGMs than the positive controls (Fig 1G). The mean vRNA quantity of in CNS tissues of vaccinated subjects was 6.1 log10 GEq/g, with the highest vRNA burden found in cervical spinal cord samples (mean 6.5 log10 GEq/g, range 5.5 – 6.9 log10 GEq/g). In unvaccinated AGMs, large quantities of vRNA were observed in all CNS tissues, averaging 10.4 log10 GEq/g (range 8.8 – 11.2 log10 GEq/g). Unlike the vaccinated subjects, the greatest mean v RNA level in positive control CNS tissues was identified in the brain stem, reaching 10.9 log10 GEq/g. Urogenital tract tissues (kidney, urinary bladder, gonad, and uterus/prostate) were sampled for HeV-g1 genomic RNA (Fig 1G). No vaccinated animals had HeV-g1 vRNA above the assay limit of detection in the kidneys; however, vRNA was detected in the urinary bladder of n = 4 subjects (mean 5.3 log10 GEq/g) and n = 5 subjects had detectable HeV-g1 genomes in gonad tissues (mean 6.5 log10 GEq/g). HeV-g1 vRNA was detected in the kidneys of control subjects and ranged from 8.8 – 11.4 log10 GEq/g, which was the greatest vRNA burden in all urogenital tissues sampled from positive control subjects.

### Gross and histopathological findings of G*rVSV∆G-NiV-G vaccinated and positive control AGMs

All five AGMs vaccinated with G*rVSV∆G-NiV-G and challenged with HeV-g1 survived to the predetermined study end point, 35 DPI (NiVG-3, NiVG-2, NiVG-4, NiVG-1, NiVG-5). No lesions indicative of fulminate HeV-g1 disease were noted at necropsy in survivors. Congestion of lungs and meninges were the most cited with NiVG-3 also having a severe incidental urocystitis, urethritis and associated urine scald dermatitis of the preputial skin. All three AGMs that served as in study positive controls succumbed to disease and were euthanized between 6 and 7 DPI (HeV-C3, HeV-C2, HeV-C1). The most severe and common lesions involved the respiratory, central nervous, and lymphoid systems. AGMs displayed moderate to marked interstitial pneumonia with fibrinous pleuritis and pleura effusion (HeV-C1, HeV-C3), congestion of the meninges (HeV-C1, HeV-C3, HeV-C2), splenopathy (HeV-C1, HeV-C2), and lymphadenomegaly (HeV-C1). No other appreciable gross lesions were noted.

Inflammatory lesions and associated IHC positivity for henipavirus N antigen were noted in at least one of the representative tissues from the unvaccinated controls. These lesions included disruption of the normal splenic follicle architecture accompanied by disruption of germinal centers and IHC labeling for HeV antigen in mononuclear cells within the white pulp (Fig 2A). Congestion of the glomerular tufts in the kidney with occasional endothelial syncytial cell and IHC positivity of the endothelium were observed (Fig 2B). Pulmonary inflammation coincided with IHC labeling of the alveolar septa, mononuclear cells, and endothelium (Fig 2C). Damage to the lungs was severe and prominent findings were expansion of alveolar septae with mixed inflammation and locally extensive areas of alveolar necrosis accompanied by fibrinaceous edema containing increased numbers of alveolar macrophages and erythrocytes. Focal, extensively vacuolated neuronal necrotic plaques with neuronal drop out was present and associated with IHC labeling of neurons and supportive cells in the temporal lobe in the brain of HeV-C3 (Fig 2D). IHC positive neurons were also identified in the brainstem of 2/3 positive controls (HeV-C3, HeV-C1). No other appreciable lesions or IHC labeling were identified.

**Fig 2:**
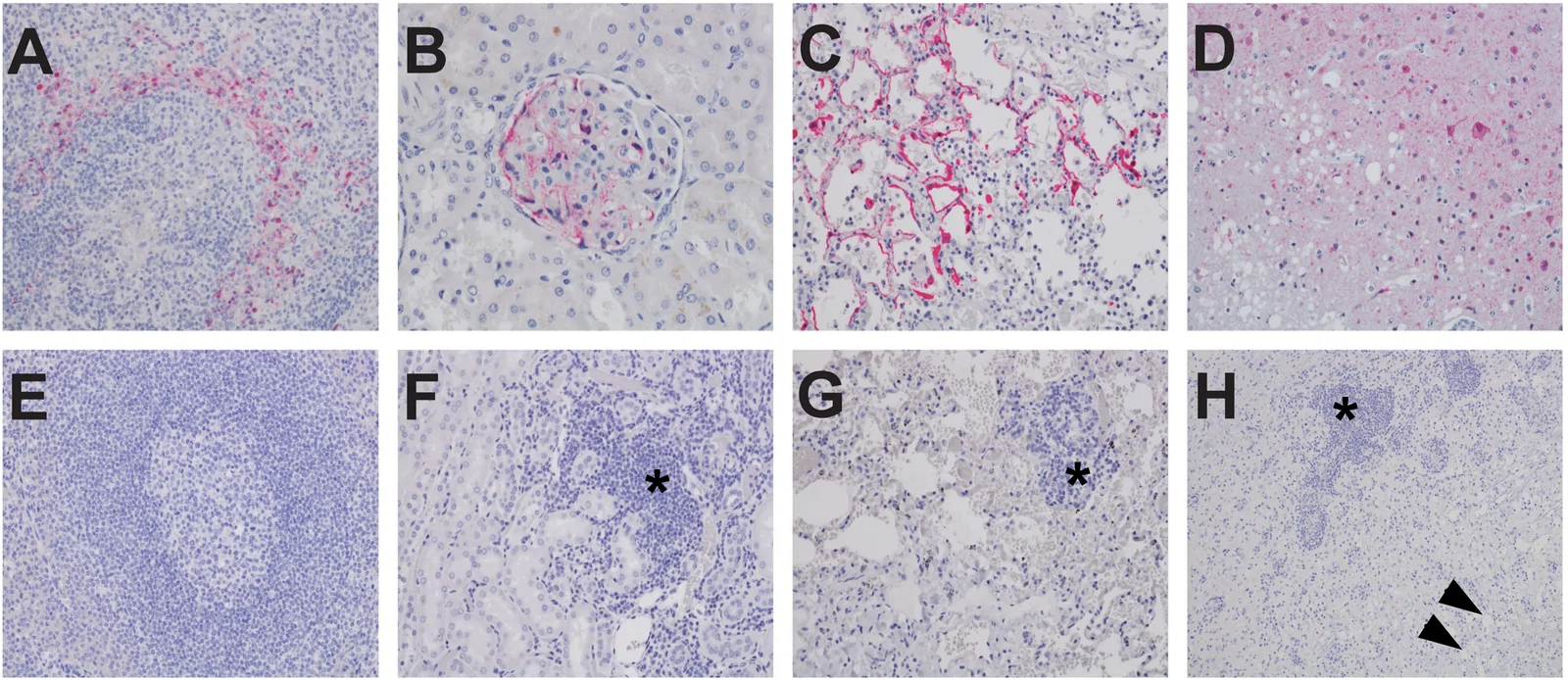
Representative immunohistochemistry (IHC) labeling for anti-henipavirus N antigen and histopathologic lesions of vaccinated and unvaccinated control AGMs. Unvaccinated control AGMs (**A-D**) and G*rVSV∆G-NiV-G vaccinated AGMs challenged with HeV-g1 (E-H). (A) IHC, spleen, 20x, HeV-C1. IHC positive (red) mononuclear cells in the white pulp of the spleen. (B) IHC, kidney, 40x, HeV-C3. IHC positive (red) endothelium of the renal glomerular tuft. (C) IHC, lung, 10x, HeV-C2. IHC positive (red) alveolar septal walls and alveolar macrophages of the lung. (D) IHC, brain (temporal lobe) 20x, HeV-C3. IHC positive (red) neurons and support cells associated with an extensive vacuolated neuronal necrotic plaque in the brain. (E) IHC, spleen, 20x, NiVG-2. No appreciable IHC labeling. (F) IHC, Kidney, 20x, NiVG-2. No appreciable labeling associated with lymphocytic interstitial infiltrates (asterisk) in the renal cortex. (G) IHC, lung, 20x, NiVG-1. No appreciable IHC labeling associated with lymphocytic interstitial infiltrates (asterisk) in the lung. (H) Brainstem, 10x, NiVG-5. No appreciable IHC labeling associated with mild vacuolation of the neuroparenchyma (arrows) and lymphocytic perivascular cuffs (asterisk) in the brainstem.

Histologic evaluation of representative tissues from the five vaccinated AGMs at the study endpoint identified inflammation in several tissues including the lung (2/5), kidney (3/5), and brain (1/5). Normal germinal center architecture was observed (Fig 2E). Minimal lymphocytic infiltrates were identified in the renal cortex of NiVG-1, NiVG-2, and NiVG-5 (Fig 2F) and the lungs of NiVG-1 and NiVG-4 (Fig 2G). Lymphocytic perivascular cuffs with mild vacuolation of the neuroparenchyma were observed in NiVG-5 (Fig 2H). No other appreciable lesions were noted histologically and no associated immunohistochemistry (IHC) labeling for anti-henipavirus N antigen was identified in the G*rVSV∆G-NiV-G vaccinated AGMs.

### Humoral and cell-mediated immune responses of G*rVSV∆G-NiV-G immunized AGMs

Components of the humoral (circulating neutralizing antibodies and total anti-HeV IgG) and cell-mediated immune response to HeV-g1 challenge were assessed by serology and targeted transcriptomics, respectively. The G*rVSV∆G-NiV-G vaccinated AGMs had minimal detectable serum IgG antibodies reactive to HeV (G) at study day 0, with reciprocal titers ranging from 1:100 – 1:400 (Fig 3A). Following challenge, anti-HeV-IgG titers rose starting at 7 DPI and peaked at 10 and 14 DPI to titers ranging from 1:51,200–1:204,800 Fig 3A). The detection of relatively low binding antibody titers at challenge corresponded to minimal neutralizing antibody responses to authentic HeV-g1 as determined by plaque reduction neutralization tests (PRNT). A 50% PRNT titer (PRNT50) against HeV-g1 was not detected at the day of challenge (1:20 dilution), and the mean percent-reduction of plaques across all vaccinated subjects at challenge was ~ 12% (Fig 3B). As was seen with in the total anti-HeV-IgG titers determined by ELISA moderate increases in plasma neutralization of HeV-g1 were observed in timepoints following challenge, starting at 7 DPI. By 10 DPI, all n = 5 G*rVSV∆G-NiV-G vaccinated subjects had developed plasma PRNT50 titers against HeV-g1 ranging from 1:160 – 1:1,240, with maximum PRNT50 neutralization titers of 1:640 – 1:2,560 (mean 1:1,408). Unvaccinated controls did not develop detectable PRNT50 neutralization titers at any point throughout the study. Conversely, all vaccinated AGMs developed neutralizing antibody titers against NiV-B, with PRNT50 titers ranging from 1:40 – 1:80 reciprocal plasma dilution at 0 DPI. By 14 DPI, PRNT50 titers to NiV-B peaked from 1:1,280 – 1:10,420 (Fig 2 in S1 Appendix).

**Fig 3:**
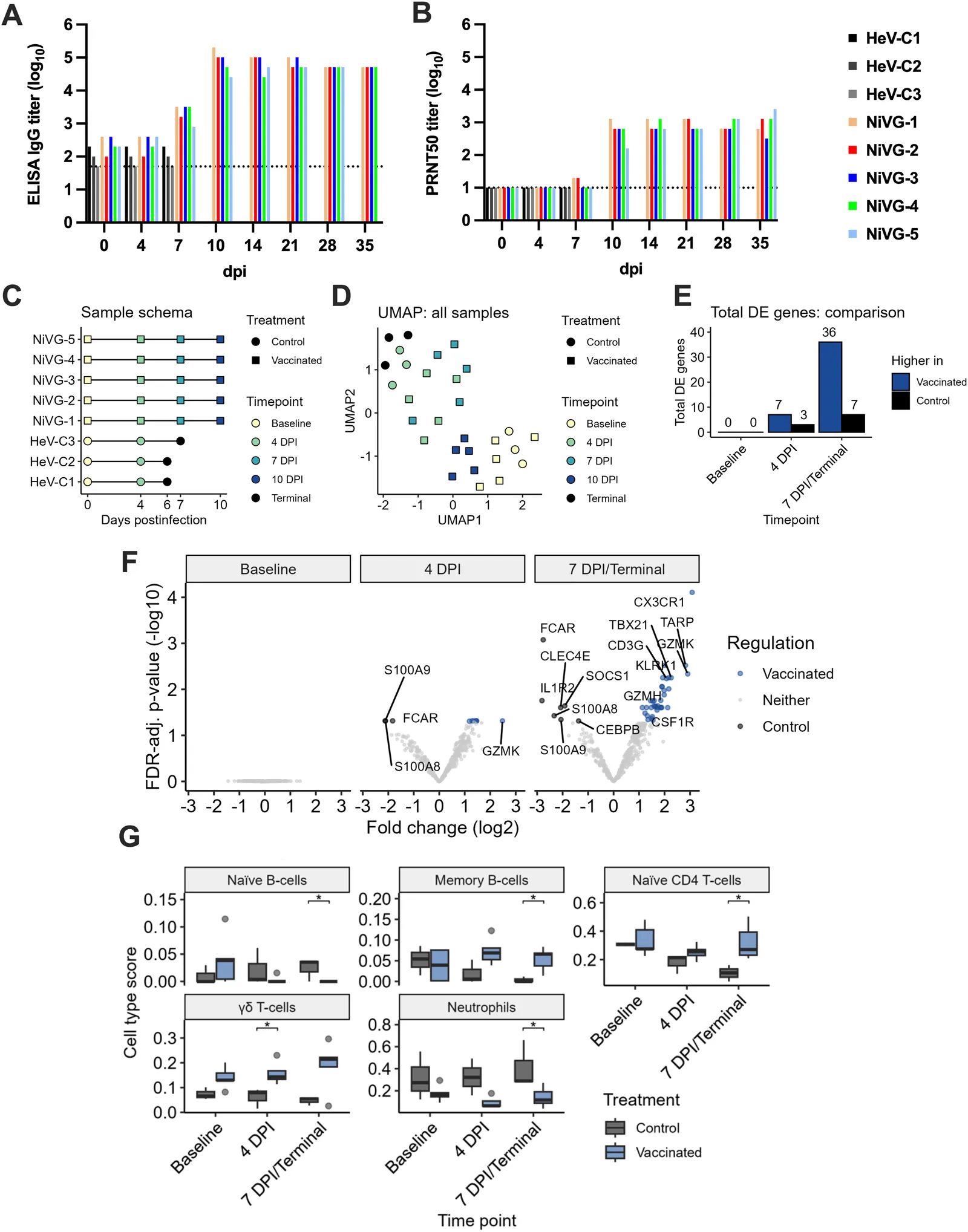
Humoral immune and transcriptomic response to G*rVSV∆G-NiV-G vaccination and HeV-g1 challenge. (A) Reciprocal endpoint anti-HeV (G) IgG antibody titers in serum samples collected from vaccinated and positive control AGMs as determined by ELISA from 0 to 35 days post-HeV-g1 challenge. The dotted line indicates the minimum sample dilution assayed (1:50). Samples with no detectable reactive antibody titer are plotted at y = 50, below the dotted line. (**B**) 50% plaque reduction neutralizing antibody titers (PRNT50) against HeV-g1 from plasma specimens of vaccinated and unvaccinated control AGMs. The last reciprocal antibody dilution where at least 50% of plaques were neutralized as compared to controls are plotted. The dotted line indicates the minimum sample dilution assayed (1:20). Samples with no detectable PRNT50 titer are plotted at y = 10. Individual Bars represent the mean of (2) technical replicates. For (**A, B**) bars represent the mean of (2) technical replicates. (C) Sampling plot of whole blood specimens analyzed by targeted transcriptomics. (D) UMAP dimension reduction plot of whole blood samples. (E) Bar plot of significant differentially expressed genes between G*rVSV∆G-NiV-G vaccinated subjects and positive controls at baseline, 4 DPI, and 7/terminal DPI. (F) Volcano plots of significant differentially expressed genes between vaccinated and positive control subjects at specified timepoints. Significance testing of log_2_ transformed fold change values was performed via Benjamini–Hochberg-corrected false discovery rate approach, -log_10_ transformed p-values are reported. (G) Differential Cell Quantification (DCQ) of inferred cell populations in vaccinated and control subjects at selected timepoints. Significant differences in cell type scores were determined via Mann-Whitney U-test.

We performed targeted transcriptomics on whole blood sampled from all AGMs in the vaccine study to investigate whether cell-mediated immunity could be implicated in the protection afforded by G*rVSV∆G-NiV-G, given the absence of strong neutralizing antibody titers at the time of challenge. Early timepoints post-challenge were specifically selected for analysis, and naïve clustering via Uniform Manifold Approximation (UMAP) revealed that all vaccinated and control samples cluster together at 0 DPI suggesting minimal differences were present in the transcriptomic profiles at baseline (Fig 3C). Transcriptomic changes became evident starting at 4 DPI and diverged greatly at 7 DPI/terminal timepoints between the vaccinated and control subjects (Fig 3D). The early response (4 DPI) to HeV-g1 challenge in G*rVSV∆G-NiV-G vaccinated AGMs was characterized by enrichment of transcripts associated with the innate immune response, primarily ISGs including *OASL, CXCL10, MX1,* and *IFIT1* (Fig 3 in S1 Appendix). When compared with positive control subjects, *GZMK* was significantly elevated starting at 4 DPI followed by other transcriptomic signatures of a mounting T- and NK-cell driven response at 7 DPI, including *CD3G, GZMH, KLRK1, CD8A* (Fig 3E). At this 7 DPI/terminal timepoint, controls exhibited upregulated transcripts associated primarily with a nonspecific and uncontrolled myeloid response *CEBPB, IL1R2, S100A8, S100A9* (Fig 3E). We performed Differential Cell Quantification (DCQ) analysis of the transcriptomic data utilizing the CIBERSORTx algorithm to extrapolate the potential cell populations in circulation (Fig 3F). Compared to positive controls, the vaccinated subjects exhibited increased cell-type scores for gamma-delta T-cells, memory B-cells, and naïve CD4 + T-cells. Higher cell-type scores were observed for neutrophils in samples collected from positive controls at the 7 DPI/terminal comparison (Fig 3F).

### Asymmetric binding and neutralizing antibody responses to NiV and HeV are replicated in Syrian golden hamsters

In the AGM study, a single-dose of the G*rVSV∆G-NiV-G vaccine did not produce substantial neutralizing antibody titers by the time of challenge (28 days post-vaccination). In combination with the targeted transcriptomics performed on whole blood specimens collected at timepoints following HeV-g1 infection, these data suggest that T-cells may play a role in G*rVSV∆G-NiV-G mediated protection against heterologous challenge. To address these findings, we examined the protective capacity of the humoral response to G*rVSV∆G-NiV-G vaccination in isolation of T- and B-cell memory through passive immunization studies in SGHs. Antiserum for passive transfer was obtained by vaccinating groups of n = 15 SGHs with 1.0E7 PFU of G*rVSV∆G-NiV-G or G*rVSV∆G-GFP and serum was collected at 28 days post-vaccination. Total anti-HeV and anti-NiV (G) specific IgG and plaque neutralization capacity of serum was assessed for individuals and a serum pool consisting of all n = 15 vaccinated subjects per group.

Serum IgG antibodies reactive to HeV or NiV (G) glycoprotein were determined by ELISA (Fig 4A). The mean reciprocal IgG antibody titer to HeV (G) was 1:337 (range <1:200 – 1:800) and 1:3227 to NiV G (range 1:400 – 1:6400). The anti-G*rVSV∆G-NiV-G pool had an IgG antibody titer to NiV of 1:12,800 and 1:1600 to HeV. No G*rVSV∆G-GFP vaccinated subjects had detectable IgG antibody titers to HeV or NiV (G) antigen by ELISA.

**Fig 4:**
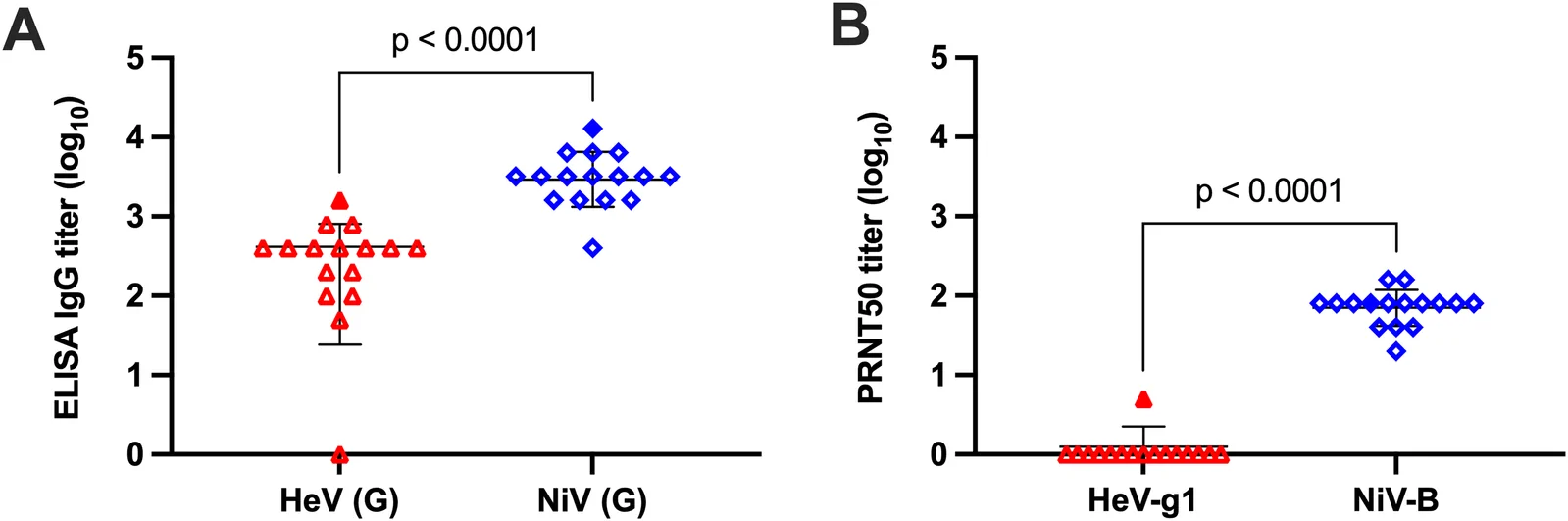
Characterization of binding and neutralizing antibodies in SGHs vaccinated with G*rVSV∆G-NiV-G. (A) Reciprocal endpoint titers of anti-HeV G (red triangles) and anti-NiV G (blue diamonds) IgG in serum of G*rVSV∆G-NiV-G vaccinated SGHs as determined by indirect ELISA. Endpoint titers are reported as the log10 last positive reciprocal dilution. (B) PRNT50 neutralizing antibody titers to HeV-g1 (red triangles) and NiV-B (blue diamonds) of G*rVSV∆G-NiV-G vaccinated SGHs. For (**A,B**) individual points represent the mean of (2) technical replicates, solid points indicate the G*rVSV∆G-NiV-G antiserum pool, and samples with no detectable binding IgG titer or PRNT50 titer are plotted at a value of 1. Horizontal lines indicate the mean, error bars indicate ±SD. Statistically significant differences between endpoint ELISA and PRNT50 titers were determined by a Wilcoxon matched-pairs signed rank test.

Neutralizing antibodies were assessed by plaque-reduction neutralization test (PRNT) against authentic HeV-g1 and NiV-B, with a 50% neutralizing titer (PRNT50) endpoint identified as the last reciprocal dilution where at least 50% of plaques were neutralized (Fig 4B). The PRNT50 titer of the serum pool against HeV was 1:5 and was 1:80 against NiV. For individual subjects, the PRNT50 titer against NiV ranged from 1:20–1:160, with a mean of ~1:80. Neutralizing antibody titers >1:10 were not detected against HeV in the G*rVSV∆G-NiV-G-vaccinated cohort samples.

### Passive transfer of G*rVSV∆G-NiV-G vaccinated antiserum reveals asymmetric survival following NiV-B or HeV-g1 challenge in Syrian golden hamsters

A single 0.5mL treatment of anti-G*rVSV∆G-NiV-G or anti-G*rVSV∆G-GFP serum pools was administered i.p. to groups of n = 8 SGHs 1 day prior to challenge with 1.0E4 PFU of NiV-B or HeV-g1 on 0 DPI, also via the IP route. Treatment with anti-G*rVSV∆G-NiV-G provided minimal protection against HeV-g1 challenge, with only a single subject surviving until the study endpoint (Fig 5A). All other anti-G*rVSV∆G-NiV-G-treated subjects challenged with HeV rapidly developed clinical signs of disease consistent with severe HeV infection including imbalance, dyspnea, hypersalivation, head-tilt, and paralysis, and were humanely euthanized by 7 DPI, with no statistical difference observed between the treatment and control groups. The single anti-G*rVSV∆G-NiV-G-treated survivor in the HeV-g1 challenge cohort did exhibit minor clinical signs of disease (rough coat, hunched posture) on 5 and 6 DPI but returned to baseline by 7 DPI. Conversely, all anti-G*rVSV∆G-NiV-G-treated subjects challenged with NiV-B survived until the study endpoint at 28 DPI without developing clinical signs of disease (Fig 5A). A total of n = 7/8 anti-G*rVSV∆G-GFP controls developed severe clinical signs of NiV-B disease (dyspnea, ataxia, tremors, head-tilt, imbalance, paralysis) and were humanely euthanized by 16 DPI; however, a single control subject survived until the study endpoint but did not exhibit clinical signs of NiV-B disease. All subjects challenged with HeV except the single survivor rapidly lost weight starting at 5 DPI and became hypothermic (Fig 5B,5C). All anti-G*rVSV∆G-NiV-G-treated, NiV-B-challenged subjects gained weight throughout the course of the study and did not develop fever or hypothermia.

**Fig 5:**
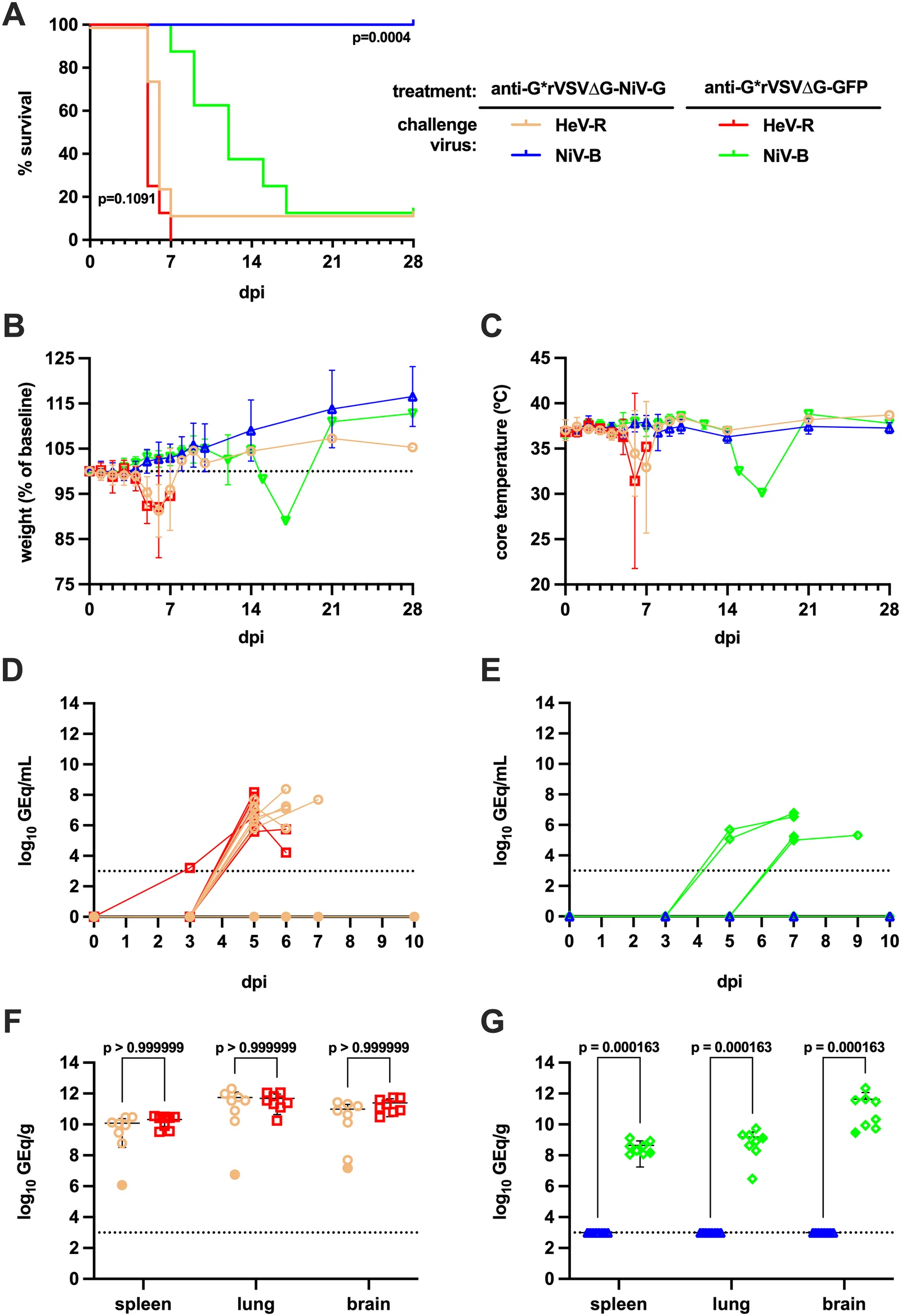
Survival, clinical disease, and viral load of SGHs treated with anti-G*rVSV∆G-NiV-G or anti-G*rVSV∆G-GFP antiserum following challenge with HeV-g1 or NiV-B. (A) Kaplan-Meier survival curve of anti-G*rVSV∆G-NiV-G and anti-G*rVSV∆G-GFP treated hamsters challenged with HeV-g1 or NiV-B. Statistically significant differences in survival curves between treatment and positive control groups challenged with HeV-g1 or NiV-B are displayed as exact p-values. (B) Percent weight change from baseline. (C) Temperature change from baseline (°C). For **B, C** points represent the mean of all subjects and error bars indicate standard deviation. (D) HeV-g1 vRNA detected in oral swabs as determined by qRT-PCR. (E) NiV-B vRNA detected in oral swabs as determined by qRT-PCR. (**F**) qRT-PCR detection of HeV-g1 vRNA in tissue specimens of positive control and anti-G*rVSV∆G-NiV-G treated SGHs. (G) NiV-B vRNA detected by qRT-PCR in tissue samples of specifically treated and anti-G*rVSV∆G-GFP treated SGHs. For **F,G** individual points represent the mean of two technical replicates. Horizontal lines indicate the mean of the group, error bars indicate standard deviation. Multiple Kolmogorov-Smirnov tests were performed to assess statistically significant of differences between distributions, exact p-values are displayed. For **D-G**, the dotted horizontal line indicates the LOD for the specified assay, 999 GEq per mL or per gram of tissue. Samples with no detectable vRNA or titer were plotted below the LOD (1 GEq/mL or 999 GEq/g).

### HeV-g1 and NiV-B quantities in oral swabs and tissues following challenge in passively immunized SGHs

Oral swabs were collected from anti-G*rVSV∆G-NiV-G- and anti-G*rVSV∆G-GFP-treated subjects at the time of challenge and on days 3, 5, 7, and 10 DPI as well as terminal timepoints and were assayed by qRT-PCR to quantify vRNA of HeV-g1 or NiV-B. In the HeV-g1-challenged, anti-G*rVSV∆G-NiV-G-treated group, all subjects except the single survivor had detectable HeV genome copies in oral swabs starting at 5 DPI (5.8 – 7.7 log10 GEq/mL) and peaked at terminal timepoints (Fig 5D). No significant difference (p=>0.9999) was observed between the oral swab vRNA quantity between anti-G*rVSV∆G-NiV-G and anti-G*rVSV∆G-GFP treatment groups at 5 DPI (Fig 4A in S1 Appendix). Conversely, NiV-B vRNA was not detected in the oral swabs of anti-G*rVSV∆G-NiV-G treated hamsters at any timepoint assayed (Fig 5E). Oral swabs collected from anti-G*rVSV∆G-GFP controls had detectable NiV-B RNA starting at 5 DPI (n = 2/8, 5.1 – 5.7 log10 GEq/mL) and at 7 DPI (n = 5/8, 5.0 – 6.8 log10 GEq/mL). A significant difference (p = 0.0269) was observed between peak oral swab viral genomes in oral swabs collected from anti-G*rVSV∆G-NiV-G and anti-G*rVSV∆G-GFP groups (Fig 4B in S1 Appendix).

Tissue samples were collected from all subjects at terminal timepoints or the predetermined study endpoint at 28 DPI for analysis of viral genome quantities by qRT-PCR. All subjects in the HeV-g1 challenged, anti-G*rVSV∆G-GFP treated control group had detectable vRNA in spleen (9.5 – 10.5 log10 GEq/g), lung (10.2 – 12.1 log10 GEq/g), and brain (10.5 – 11.7 log10 GEq/g) tissues (Fig 5F). HeV-g1 vRNA was also detected in all tissues sampled from subjects in the anti-G*rVSV∆G-NiV-G-treated group including spleen (6.1 – 10.5 log10 GEq/g), lung (6.7 – 12.3 log10 GEq/g), and brain (7.2 – 11.4 log10 GEq/g). The single anti-G*rVSV∆G-NiV-G survivor subject exhibited lower quantities of HeV-g1 vRNA then all other treated subjects but no significant difference between distributions was observed. NiV-B vRNA was detected in all anti-G*rVSV∆G-GFP treated controls, including the natural survivor, with 8.1 – 9.1 log10 GEq/g in the spleen, 6.5 – 9.7 log10 GEq/g in the lungs, and 9.5 – 12.3 log10 GEq/g in the brain (Fig 5G). There was no NiV-B vRNA detected in the tissues of any anti-G*rVSV∆G-NiV-G-treated subjects (LOD 2.9 log10 GEq/g), and significant differences in vRNA quantities were observed compared to the control subjects.

### Gross and histopathological lesions of anti-G*rVSV∆G-NiV-G and anti-G*rVSV∆G-GFP treated SGHs challenged with HeV-g1 or NiV-B

Tissues specimens (spleen, lung, and brain) were collected at necropsy from all subjects and evaluated histological by a board-certified veterinary pathologist blinded to treatment group. Representative photomicrographs of anti-henipavirus N antigen immunolabeling by IHC are shown in Fig 6 and severity scoring of H&E and IHC sections examined is shown in Table 1, S1 Appendix. Subjects that were humanely euthanized due to severe clinical signs of henipavirus disease had histopathological lesions consistent with henipavirus infection in the lungs, spleen, and brain that colocalized with immunolabeling for henipavirus N antigen. Pulmonary lesions for fatally infected subjects challenged with HeV-g1 or NiV-B included multifocal acute necrotizing interstitial pneumonia with vasculitis, syncytial cells, and flooding of alveolar spaces with edema, fibrin, cellular debris, and mixed inflammation consisting of lymphocytes and neutrophils. Alveolar septal walls, mononuclear cells, syncytial cells, endothelium, and smooth muscle of vessels were IHC positive (Fig 6A, 6C, 6D). Primary findings in the splenic tissue of fatally infected subjects included disruption of lymphoid architecture with mild- to marked loss of lymphocytes within the white pulp of the spleen accompanied by hypercellularity of the red pulp and IHC positivity of mononuclear and endothelial cells (arrows) (Fig 6I, 6K, 6L). Lesions within the brain of non-survivors included mild gliosis and occasional perivascular lymphocytic cuffs (arrows) (Fig 6W). IHC positive neurons, endothelium, vascular smooth muscle, and fibrous tissue of the pachymeninges were also observed (Fig 6Q, 6S, 6T, 6W). A subject in the control group that was euthanized at 17 DPI exhibited extensive IHC positivity of neurons (Fig 6W), but no immunolabeling for NiV-B antigen was observed in the lungs or spleen tissue (Fig 6G,6O). No appreciable immunolabeling or inflammatory lesions were appreciable for the anti-G*rVSV∆G-NiV-G treated survivor challenged with HeV (Fig 6B, 6J, 6R), the natural survivor in the NiV-B control group (Fig 6H,6P,6X) or the anti-G*rVSV∆G-NiV-G treated subjects challenged with NiV-B (Fig 6E, 6F, 6M, 6N, 6U, 6V).

**Fig 6:**
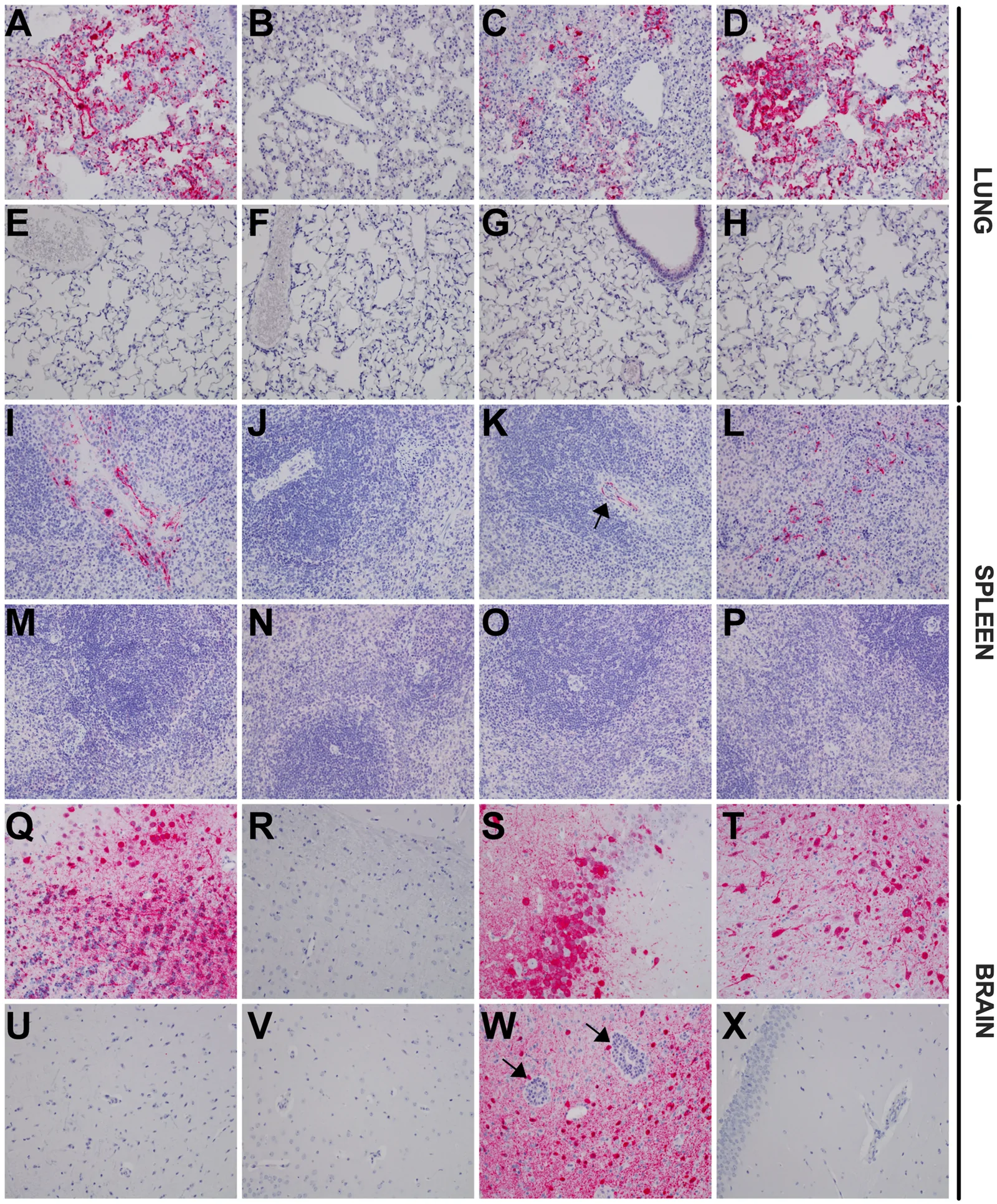
Immunohistochemistry (IHC) labeling for anti-henipavirus N antigen in representative tissues of antiserum treated SGHs. All images were captured with a 20x objective. IHC positive (red) pulmonary tissues for anti-G*rVSV∆G-NiV-G treated, HeV-g1 challenged subject 1-3 (A), anti-G*rVSV∆G-GFP treated, HeV-g1 challenged, subjects 2-1 (**C**) and 2-5 (D). Lack of IHC positivity (red) in pulmonary tissues for anti-G*rVSV∆G-NiV-G treated, HeV-g1 challenged subject 1-7 (B), anti-G*rVSV∆G-NiV-G treated, NiV-B challenged subjects 3-3 (**E**) and 3-5 (F), anti-G*rVSV∆G-GFP treated, NiV-B challenged subjects, 4-3 (**G**) and 4-7 (H). IHC positive (red) splenic tissues for Group 1, 1-3 (I), Group 2, 2-1 (K), 2-5 (L). Lack of IHC positivity (red) in splenic tissues for Group 1, 1-7 (J), Group 3, 3-3 (M), 3-5 (N), Group 4, 4-3 (O), 4-7 (P). IHC positive (red) neural tissues for Group 1, 1-3 (Q), Group 2, 2-1 (S), 2-5 (T), Group 4, 4-3 (W). Lack of IHC positivity (red) in neural tissues for Group 1, 1-7 (R), Group 3, 3-3 (U), 3-5 (V), Group 4, 4-7 (X).

## Discussion

Nipah and Hendra viruses continue to pose a significant threat to human and animal health. Although a highly efficacious veterinary vaccine is approved for use in Australia to prevent equine HeV cases, sporadic zoonotic transmission events continue to occur, primarily driven by ecological factors [46]. The shedding of infectious HeV from the *Pteropus spp.* natural reservoir presents a significant risk for HeV cases in unvaccinated horses, which can subsequently infect humans. The henipavirus F and G surface glycoproteins are primary targets of medical countermeasures, and the G glycoproteins of NiV and HeV share ~83% amino acid homology. The resulting antigenic similarity has been leveraged in multiple experimental vaccine countermeasures based on the HeV G immunogen and successfully demonstrated protection in multiple preclinical animal studies [30,43,47], and most recently, immunogenicity in results reported from the Phase I evaluation of the HeV-sG-V vaccine [48]. Previous efforts to utilize the NiV G glycoprotein in a vaccine countermeasure to protect against HeV have not resulted in uniform survival [31–33], suggesting certain vaccine platforms or adjuvant formulations may not be ideal for inducing a cross-protective response.

Given the previously demonstrated efficacy of the G*rVSV∆G-NiV-G vaccine against NiV in combination with the immunogenic properties of the G*rVSV∆G platform, we sought to determine whether this countermeasure could offer protection against closely related HeV-g1 in a single-dose, antigen-mismatched vaccine study. We found that a single, 1.0E7 PFU dose of G*rVSV∆G-NiV-G protected against lethal HeV disease with all vaccinated subjects surviving until the predetermined study endpoint at 35 DPI. However, 2/5 G*rVSV∆G-NiV-G vaccinated subjects did exhibit mild clinical signs following HeV-g1 challenge, including increased respirations and thrombocytopenia. Furthermore, HeV-g1 vRNA was detected in whole blood samples collected at acute disease timepoints (4,7, and 10 DPI) but was subsequently cleared and no replicating virus was detected in plasma. Interestingly, protection against HeV was achieved in the absence of high titers of potently neutralizing antibodies, a well-described immune correlate typically observed in protective response induced by other rVSV-vectored vaccines. We did observe transcriptomic signatures in the blood of vaccinated subjects indicative of a T- and NK-cell response which may play an important role in driving clearance of viremia and ultimately, successful cross-protection against HeV challenge with a NiV antigen.

To further investigate the contribution of HeV-neutralizing serum antibodies to the protection afforded by G*rVSV∆G-NiV-G, we conducted passive immunization studies utilizing highly sensitive SGH models of HeV-g1 and NiV-B. Vaccination of SGHs with G*rVSV∆G-NiV-G resulted in development of low serum neutralizing antibody titers to HeV-g1 at 28 days post-vaccination, similar to what was observed in the AGM experiments. ELISAs also revealed that total serum anti-HeV (G) IgG was lower than that to NiV (G). Passive transfer of anti-G*rVSV∆G-NiV-G pools 1 day prior to challenge provided essentially no protection against lethal disease and a delay in time to death was not observed for the 7/8 G*rVSV∆G-NiV-G treated subjects that succumbed to HeV disease. In contrast, the same treatment uniformly protected against antigen-matched NiV-B challenge. We have previously shown that an experimental HeV (G) antigen vaccine using the same G*rVSV∆G platform could provide single-dose protection against both HeV-g1 and NiV-B challenge in SGHs; however, passive transfer of antiserum raised in the same manner as the anti-G*rVSV∆G-NiV-G pool described in the present work provided partial, yet equivalent protection [43]. Because AGMs vaccinated with G*rVSV∆G-NiV-G were protected from severe and lethal HeV disease, it is possible that prophylaxis with a higher dose of anti-G*rVSV∆G-NiV-G serum may improve survival outcomes in the highly stringent SGH model. Future studies should be performed to more precisely examine the dose-dependent protection mediated by treatment with polyclonal antibodies raised against experimental henipavirus vaccines.

Although survival outcomes between the SGH and AGM studies were not correlated, the serology results were nearly identical. The inability of a single dose of the G*rVSV∆G-NiV-G vaccine to elicit a potent neutralizing antibody response to HeV in context of vaccine-mediated survival suggests that cross-protection by a NiV (G) immunogen requires a cell-mediated immune response which has shown to be present in human natural survivors of NiV disease [49] and required for vaccine-mediated protection against NiV in swine [50]. Other experimental vaccines have demonstrated antigen-matched protection in the absence of strong neutralizing antibody titers [51,52], but it has previously not been reported whether the suspected cell-mediated response could protect against heterologous challenge. In the present study, we isolated the humoral response to G*rVSV∆G-NiV-G through passive transfer experiments and used targeted transcriptomics to identify signatures associated with T- and NK-cells. Our findings suggest that a cell-mediated immune response may compensate for the diminished humoral responses we observed to NiV (G) immunogen, but functional studies such as adoptive transfer or immune-cell depletion should be conducted to definitively reveal the specific contribution of T-cells to vaccine-mediated protection.

In summary, we identified that a single dose of the G*rVSV∆G-NiV-G vaccine can cross-protect against HeV-g1 challenge in a gold-standard nonhuman primate model of Hendra virus disease without eliciting a potent HeV neutralizing antibody response. Specifically, we demonstrated a NiV (G) antigen in a single-cycle viral-vectored vaccine could provide complete survival against HeV-g1 challenge, which has not been previously shown. However, the mild disease observed in two of the vaccinated AGMs and failure of vaccinated antiserum as an effective prophylactic in SGHs supports that a more robust cross-protective response to NiV and HeV can be elicited by HeV (G). Given the continued spillover of NiV, HeV, and other emerging henipaviruses, development of more robust cross-protective vaccine countermeasures for these pathogenic agents should be a priority.

## Supporting information

S1 Appendix Fig 1. HeV-g1 viral genomes detected by qRT-PCR in respiratory tissues of G*rVSV∆G-NiV-G-vaccinated and unvaccinated positive control AGMs. Fig 2. Reciprocal endpoint PRNT50 neutralization titers plasma sampled from VSV-NiV-vaccinated subjects against NiV-B. Fig 3. Differentially expressed genes in G*rVSV∆G-NiV-G-vaccinated subjects across sampling timepoints post-infection as compared to 0 DPI. Fig 4. Comparison of peak viral genome equivalents detected in oral swabs collected from anti-G*rVSV∆G-NiV-G-treated SGHs following challenge with HeV-g1 or NiV-B. Table 1. Histopathology severity scores for representative tissues in anti-G*rVSV∆G-NiV-G- and anti-G*rVSV∆G-GFP-treated hamsters following challenge with HeV-g1 or NiV-B. (DOCX)

S2 Appendix Raw data corresponding to main manuscript figures. (XLSX)
