# Supplementary material for: A single-cycle, recombinant VSV platform Nipah vaccine cross-protects against Hendra virus in nonhuman primates
Source: PLoS Pathog. 2026 Sep 22;22(9):e1014646. doi: 10.1371/journal.ppat.1014646 (PMC13623150; doi:10.1371/journal.ppat.1014646)
Supplement: Supplementary file 1 [file ppat.1014646.s001.docx]

**S1 Appendix Fig 1. HeV-g1 viral genomes detected by qRT-PCR in respiratory tissues of G*rVSV∆G-NiV_B_-G-vaccinated and unvaccinated control AGMs.** Individual points represent the mean of two technical replicates. Horizontal lines indicate the mean, error bars indicate standard deviation. Dotted horizontal line indicates the LOD for the qRT-PCR assay, 999 GEq/g of tissue. Samples with no detectable vRNA or titer were plotted below the LOD (1 GEq/g). RUL, right-upper lobe; RML, right-middle lobe; RLL, right-lower lobe; LUL, left-upper lobe; LML, left-middle lobe; LLL, left-lower lobe; NasMuc, nasal mucosa.

**S1 Appendix Fig 2. Reciprocal endpoint PRNT50 neutralization titers plasma sampled from VSV-NiV-vaccinated subjects against NiV-B.** Individual colored bars represent the mean of (2) technical replicates for each subject at the specified timepoint. Samples with no PRNT50 titer at the minimum assayed dilution of 1:20 are plotted at y=10.

**S1 Appendix Fig 3. Differentially expressed genes in rVSV-NiV-vaccinated subjects across sampling timepoints post-infection as compared to 0 DPI.** Select transcripts are annotated.

**S1 Appendix Fig 4. Comparison of peak viral genome equivalents detected in oral swabs collected from anti-G*rVSV∆G-NiV-G treated SGHs following challenge with HeV-g1 or NiV-B. (A)** Peak viral RNA load detected in oral swabs of anti-G*rVSV∆G-NiV-G treated and positive control SGHs following challenge with HeV-g1. **(B)** Peak viral RNA load in oral swabs of anti-G*rVSV∆G-NiV-G treated subjects following challenge with NiV-B. Multiple Kolmogorov-Smirnov tests were performed to assess statistical significant of differences between distributions, exact p-values are displayed. Individual points represent the mean of technical replicates, horizontal lines indicate the mean, and error bars indicate standard deviation. Dotted horizontal line indicates the LOD for the specified assay, (999 GEq per mL), and samples with no detectable vRNA are plotted below the LOD at y=1 for visualization on the log axis.

**S1 Appendix Table 1: Histopathology severity scores for representative tissues in anti-G*rVSV∆G-NiV-G and anti-G*rVSV∆G-GFP-treated hamsters following challenge with HeV-g1 or NiV-B.**

|  | **anti-G*rVSV∆G-NiV-G Tx** | | | **anti-G*rVSV∆G-GFP Tx** | | | **anti-G*rVSV∆G-NiV-G Tx** | | | **anti-G*rVSV∆G-GFP Tx** | | |
| --- | --- | --- | --- | --- | --- | --- | --- | --- | --- | --- | --- | --- |
| **Virus** | **HeV-g1** | | | **HeV-g1** | | | **NiV-B** | | | **NiV-B** | | |
| **Animal ID** | 1-1 | 1-3 | 1-7 | 2-1 | 2-5 | 2-8 | 3-3 | 3-5 | 3-7 | 4-2 | 4-3 | 4-7 |
| **DPI** | 7 | 5 | 28 | 7 | 5 | 6 | 28 | 28 | 28 | 7 | 17 | 28 |
| **Spleen** | 1 1 | 1 1 | 0 0 | 1 1 | 1 1 | 1 1 | 0 0 | 0 0 | 0 0 | 0 0 | 0 0 | 0 0 |
| **Lung** | 3 3 | 3 3 | 0 0 | 2 2 | 3 3 | 2 2 | 0 0 | 0 0 | 0 0 | 2 0 | 0 0 | 0 0 |
| **Brain** | 1 3 | 1 3 | 0 0 | 1 3 | 1 3 | 1 3 | 0 0 | 0 0 | 0 0 | 0 0 | 2 3 | 0 0 |
| **First score indicates severity of H&E slide.**  **Second score indicates severity of IHC slide.**  **1 = 1-25% of examined tissues with lesions or % IHC labeling, only 1 cell type that is IHC+.**  **2 = 26-50% of examined tissues with lesions and/or necrosis or % IHC labeling.**  **3 = 51-75% of examined tissues with lesions and/or necrosis or % IHC labeling.**  **4 = 76-100% of examined tissues with lesions and/or necrosis or % IHC labeling.** | | | | | | | | | | | | |
